# “*Probably a Little Bit of a Hill to Climb*”: A qualitative study of emergency department providers’ perceptions of nonpharmacological pain treatment

**DOI:** 10.1371/journal.pone.0350266

**Published:** 2026-06-01

**Authors:** Rogelio A. Coronado, David G. Schlundt, Kristin R. Archer, Kemberlee R. Bonnet, Carrie E. Brintz, Tyler Toledo, Vivek P. Joseph, Ruth Q. Wolever, Catherine S. Hobbs, Mira P. Patel, Sean P. Collins, Alan B. Storrow

**Affiliations:** 1 Department of Orthopaedic Surgery, Vanderbilt University Medical Center, Nashville, Tennessee, United States of America; 2 Vanderbilt Center for Musculoskeletal Research, Vanderbilt University Medical Center, Nashville, Tennessee, United States of America; 3 Department of Physical Medicine and Rehabilitation, Vanderbilt University Medical Center, Nashville, Tennessee, United States of America; 4 Osher Center for Integrative Health, Vanderbilt University Medical Center, Nashville, Tennessee, United States of America; 5 Department of Psychology, Vanderbilt University, Nashville, Tennessee, United States of America; 6 Department of Anesthesiology, Vanderbilt University Medical Center, Nashville, Tennessee, United States of America; 7 Veteran Administration Tennessee Valley VA Health Care System, Nashville, Tennessee, United States of America; 8 Department of Emergency Medicine, Vanderbilt University Medical Center, Nashville, Tennessee, United States of America; 9 Veteran Administration Tennessee Valley VA Health Care System Geriatric Research Education Clinical Center (GRECC), and the Veterans’ Wellbeing through Innovation, Systems Science and Experience in Learning Health Systems (VETWISE-LHS), Nashville, Tennessee, United States of America; Edo State University Uzairue, NIGERIA

## Abstract

**Background:**

Nonpharmacological strategies are advocated as evidence-based treatment options for pain, yet these are rarely offered within the emergency department (ED) setting. Understanding how ED providers perceive these strategies can guide implementation efforts.

**Objective:**

The objective of this study was to qualitatively examine ED provider perceptions of conventional and complementary nonpharmacological pain strategies.

**Methods:**

Nine ED physicians from a single academic medical center completed a semi-structured interview conducted by a trained qualitative researcher. The interview focused on the provider’s current pain management approach and perceived benefits, barriers, and facilitators of nonpharmacological pain treatments. Each interview was audio-recorded, transcribed verbatim, and analyzed using an iterative deductive–inductive approach. Findings were organized into themes and subthemes to inform a conceptual model of nonpharmacological intervention implementation.

**Results:**

Six major themes emerged: 1) institutional context around intervention implementation, 2) professional beliefs about nonpharmacological pain interventions, 3) patient characteristics as a modifying factor, 4) intervention characteristics as a modifying factor, 5) process of implementation, and 6) engagement. Providers acknowledged benefits of nonpharmacological strategies, particularly for patients with chronic pain or history of opioid use. However, perceived barriers included negative patient perceptions of mind-body therapies, minimal ED provider training or education, limited time or care coordination support, and lack of physical space. Possible facilitators for integration included provider education, leadership support, and intervention tailoring.

**Conclusion:**

ED providers recognize the potential value of nonpharmacological pain treatment strategies. However, both broad healthcare and ED-specific barriers to implementation may limit routine use in the ED. Future efforts for improving pain management in the ED should identify strategies to address implementation barriers of evidence-based nonpharmacological interventions.

## Introduction

Pain is the leading reason people visit the emergency department (ED), with up to 70% of visits attributed to a traumatic or non-traumatic painful condition [1–3]. Pain is often located in the low back, abdomen/pelvis, or head, and musculoskeletal in origin [4,5]. The severity, urgency, and acuity of pain presentations in the ED are variable. Most patients report high levels of acute pain [3,6]; however, estimates suggest chronic pain accounts for 10–19% of all ED visits [5,7,8].

Pharmacological interventions are the most commonly used strategy for pain control in the ED, with opioids administered or prescribed in up to 40% of visits for pain conditions such as low back pain [6,9–12]. While offering short-term relief [13], opioid provision has limited benefit for long-term efficacy and must be weighed against concerns for side effects, misuse, prolonged use or dependency, and their association with an increase in return ED visits [13–19]. Clinical practice guidelines and best practices for acute and chronic pain management recommend nonpharmacological strategies as first-line options for multimodal care [20,21]. However, these guidelines and practice patterns are not easily transferable to the ED setting [22].

The biopsychosocial model relating to pain generally posits that biological, psychological, and social factors individually and collectively influence pain onset, progression, and recovery [23]. Nonpharmacological interventions are non-drug, non-invasive interventions including physical, psychological, behavioral, social, lifestyle, spiritual, and environmental strategies that aim to influence health or participation in health-related activities or prevent disease [24]. Nonpharmacological interventions targeting biopsychosocial pain factors encompass a range of mind-and body-based therapies that are efficacious in reducing pain and improving function [25]. Examples of these interventions include physical therapy, acupuncture, spinal manipulation, and counseling. Recent ED patient survey studies have found that over 90% of patients with musculoskeletal pain are willing to try nonpharmacological strategies and over 70% of patients are willing to try modalities such as physical therapy or psychosocial interventions [26,27]. However, less than 5% of ED patients receive what can be considered an evidence-based nonpharmacological option (e.g., advice to stay active) [28].

Given the unique ED environment with complex patient presentations, pressing time demand, and constrained resources, understanding provider perceptions on integration of nonpharmacological approaches is critical. This qualitative study addresses an important knowledge gap on barriers and facilitators influencing ED provider use and patient engagement with conventional and complementary nonpharmacological interventions.

## Methods

### Study design

This was a cross-sectional qualitative study examining the perceptions of ED physicians who triage, evaluate, and treat patients with acute or chronic pain. We conducted one-on-one semi-structured interviews between a trained qualitative researcher and ED physicians. The semi-structured interview guide was developed with input from ED physicians, musculoskeletal pain researchers, and qualitative experts from the Vanderbilt University Qualitative Research Core (VU-QRC). This study was approved by the Institutional Review Board (IRB) of Vanderbilt University Medical Center. Recruitment occurred from April 29, 2022 to June 24, 2022. IRB-approved electronic informed consent was obtained from each participant. The reporting of study findings followed the Consolidated Criteria for Reporting Qualitative Research guidelines (COREQ) [29].

### Setting and participants

This study was conducted at an academic medical center with a Level 1 trauma center in the state of Tennessee. Approximately 85,000 patients present to the medical center’s ED annually. A convenience sample of fifteen full-time ED physician providers were recruited by email. Six providers were unavailable to participate. Nine providers (N (%) female = 1 (11%), median years of experience = 10.5 years) were enrolled and completed the study. The VU-QRC agreed that saturation had been reached with this sample size when there were no indications of new themes emerging in the final interviews [30].

### Interviews

Individual, semi-structured interviews lasting from 30 to 45 minutes in duration were conducted via telephone with a trained qualitative researcher (K.B., MA in Social Psychology, VU-QRC Senior Research Manager, female, 13 years of qualitative research experience). The qualitative researcher holds a position that is external to the ED and independent of clinical hierarchy and has no clinical background with nonpharmacological interventions or emergency medicine. She has collaborated previously with one interviewee for research. Participants were informed that the goal of the interview was to know more about provider’s thoughts and opinions on nonpharmacological pain interventions that can be used in the ED as complements or alternatives to both non-opioid and opioid medication. A formal definition of nonpharmacological interventions was not provided to interviewees, however, examples offered by the qualitative researcher were physical therapy, counseling, acupuncture, and mindfulness. Participants were also free to introduce or discuss their own examples.

The interview guide included open-ended questions pertaining to 1) current pain management approaches; 2) knowledge and understanding of nonpharmacological pain management strategies; and 3) barriers and facilitators to implementing nonpharmacological pain management interventions in the ED setting (S1 File). Follow-up questions were asked for clarity purposes and to facilitate detailed discussion. Interviews were audio recorded and transcribed verbatim using an IRB-approved transcription service (rev.com). Transcripts were not reviewed by participants since they were recorded and transcribed verbatim.

### Data coding and analysis

Qualitative data coding and analysis was managed by the VU-QRC, led by a PhD-level psychologist (D.S.). A hierarchical coding system (S2 File) was developed and refined using the interview guide, the Consolidated Framework for Implementation Research (CFIR), and a preliminary review of the transcripts. The coding system included major categories around discussion of: 1) pain management approach; 2) nonpharmacological intervention characteristics; 3) hospital organizational setting; 4) outer setting (e.g., external environment to the hospital setting); 5) provider attitudes, beliefs, and behavior; 6) patient factors; 7) all aspects of communication (e.g., mode, quality, and between various persons); 8) barriers and facilitators to nonpharmacological pain treatment; 9) specific examples; 10) process needed for implementation; 11) system-level suggestions and needs for implementation; 12) setting context (e.g., specifics about emergency department); 13) provider(s) or health team member(s) involved; 14) provider practice/work experience; 15) world events; 16) change over time; and 17) notable quotes. Major categories were further divided from two to 10 subcategories, with some subcategories having additional levels of hierarchical division. Definitions and rules were written for the use of coding categories.

Two experienced VU-QRC qualitative data analysts first established reliability in using the coding system on two transcripts. Coding of each transcript was compared, and any discrepancies resolved through reconciliation discussion meetings [31]. The coders then divided and independently coded the remaining seven transcripts. Each statement was treated as a separate quote and could be assigned up to 15 different codes. Transcripts were combined and sorted by code. The transcripts, quotations, and codes were managed using Microsoft Excel, Office 365 (Microsoft Corporation, Redmond, WA) and IBM SPSS Statistics for Windows, Version 28 (IBM Corporation, Armonk, NY). Artificial intelligence was not used at any step in the process.

We used an iterative inductive/deductive approach to qualitative analysis, resulting in a conceptual framework illustrating perceived benefits, barriers, and facilitators to implementing nonpharmacological pain management services within the ED setting [32–34]. Inductively, we sorted the quotes by coding category and used the sorted quotes to identify higher order themes and relationships between themes. Deductively, we were guided by CFIR due to the focus on treatment implementation and the biopsychosocial model as patient factors stem from biological, psychological, and social domains [35–37]. We were also guided by the Social Ecological Framework [38,39], especially as the framework helped understand environmental factors that contribute to the patients’ attitudes about or ability to participate in nonpharmacological pain management strategies. The framework development process was iterative in that the conceptual framework is theoretically informed, while the specific framework content is derived and revised from the qualitative data. The conceptual framework was iteratively discussed, revised, and refined by the entire study team, creating a synthesis of deductive and inductive themes to most clearly express these data. Any discrepancies were resolved by discussion and consensus.

## Results

### Conceptual framework

**Fig 1:**
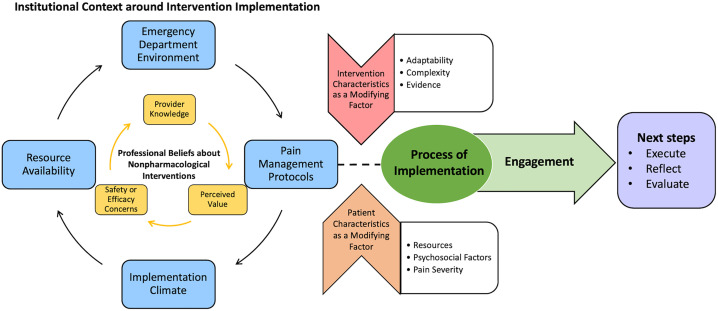
Framework for implementation of nonpharmacological pain treatments in the emergency department. The figure describes the organizational, individual, and contextual factors that influence nonpharmacological intervention implementation. Institutional context and provider belief factors influence the process of implementation, while the characteristics of the intervention and patient can moderate the extent to which an intervention is implemented. Successful implementation requires personnel engagement prior to evaluation.

Fig 1 graphically displays a conceptual framework informed by themes and subthemes (Table 1). The framework demonstrates that there is an interaction between institutional context and professional beliefs that influence implementation processes, modified by characteristics of the patient and intervention. On the left side of the framework, embedded circles represent the influence of the ED environment, pain management protocols, implementation climate, and resources availability on providers’ professional beliefs. Provider beliefs that influence implementation are intervention specific and stem from the provider’s knowledge of a nonpharmacological pain intervention, the perceived value of the intervention, and related concerns about safety or efficacy. Intervention characteristics and patient environment were identified as modifying factors that can influence the implementation processes. Relevant patient factors include the patient’s biopsychosocial characteristics and pain severity, while relevant intervention factors include the adaptability, complexity, and existing evidence supporting the intervention. Together, these modifying factors influence the implementation process, including the development of plans and engaging the appropriate individuals for successful implementation. Finally, the intervention is executed, with ongoing reflection and evaluation.

**Table 1:** Themes and sub-themes.

Themes	Sub-themes
Institutional context around intervention implementation	• Emergency department environment • Pain management protocols • Implementation climate • Resource availability
Professional beliefs about nonpharmacological pain interventions	• Provider knowledge • Perceived value • Safety or efficacy concerns
Patient characteristics as a modifying factor	• Resources • Psychosocial factors • Pain severity
Intervention characteristics as a modifying factor	• Adaptability • Complexity • Evidence
Process of implementation	• Planning for workflow integration • Provider education
Engagement	• Provider-level engagement • Support from leadership

In the following sections, we discuss each theme and subtheme of the conceptual framework and Table 1, with supporting italicized quotes from ED providers (participant number identifies source of the quote) in the text and respective tables.

### Institutional context around intervention implementation

Table 2 contains illustrative quotes related to institutional context. Providers characterized the ED as a safety net for both acute and chronic pain and at times, a primary or specialty care setting. Pain is viewed as a common chief complaint, with multiple pain-related interactions occurring during a typical shift. Patient placement into treatment areas within the ED setting, quick decision making, treatment team collaboration and coordination, and care transitions are common elements of the ED workflow. For patients with acute or chronic pain, decisions about care are often made within a designated “fast track” area.

**Table 2:** Institutional context around intervention implementation.

Emergency department environment	
Description of pain encounters	*“…I would say probably half of the patients I see have a pain related complaint… about a quarter of folks… I'd say have some kind of ongoing pain complaint that may be subacute to chronic.” (P9)*
Decision-making	*“… I think as an emergency physician, you frequently assess the patient and determine really quickly...kind of put them in the buckets like absolutely getting admitted, definitely going home.” (P1)* *“They go to our little fast track area that is six recliner chairs in the same room… And it's a way for us to be able to rapidly see them and then make sure that everything is okay, get X rays, whatever, and discharge them in the quickest possible manner...(P3).”*
Patient population	*“We're the safety net of the healthcare system… not only acute problems, but chronic problems because these patients have oftentimes nowhere to turn to… a lot of these patients may not have insurance, or if they're underinsured, they don't have a provider that they can go to…” (P8)*
**Pain management protocols**	
Opioid policies and guidelines	*“We do have a policy for no opiates, which I'm glad that we have that policy to help us discuss that with the patients, because they're frequently not happy about that (P2).”* *“It'd be nice to have guidelines if they're available because I don't know of any general guidelines for emergency departments… Acutely, I try to avoid narcotics as much as possible. In patients who have acute exacerbations of chronic pain, I generally avoid narcotics or opioids almost all the time.” (P8)*
Multimodal approaches for pain	*“...if someone comes in with an obvious broken femur, then I go right to opiates. To me that's an acute, severe pain, and pain control is really important (P2).”* *“For sciatica, for example, I'll use NSAIDs, acetaminophen, and then also discuss with them about their management at home…, I try to get them some transient improvement, not resolution in their pain, to help them understand also what the appropriate expectations are. We're not going to get this gone magically, that opioids are not going to improve their pain, in that, we'll also have to do things like stretching, physical therapy, and topicals to be able to help with that.” (P5)* *“I'm trying to figure out if there's a way that I can use a non-opioid-based approach. Now, if they've been on chronic opioids, the likelihood that I'm going to be able to do so is low, so I'll try to have a discussion with the patient about doing so, and I can quickly get a sense of whether or not the patient is even going to be [open] to that (P5).”*
**Implementation climate**	
Compatibility	*“Well, I mean, treating pain is complex… I do think counseling can work really well for people. I don't really see how that would fit into our workflow and emergency department.” (P1)* *“We're always on a timer kind of thing…. There is time to have an intervention of some sort if it was a helpful one. It's just that once their disposition has been made, then they need to leave, because there's so many that need to be there.” (P4)*
Receptiveness	*“I've had discussions with people about physical therapy within the emergency department. I think people would find that useful.” (P1)* *“… We're running out of options for some of those patients, so having some additional person come talk specifically about pain is a great idea.” (P6)*
**Resource availability**	
Physical space	*“You can't do acupuncture in a hallway. God forbid somebody falls on top of somebody who's got a bunch of needles on their back…The resources outside of having someone who's qualified to do it and make sure that it happens are less in terms of materials and really space. Real estate is the biggest issue.” (P9)*
Time	*“I think that if things (nonpharmacologic treatments) took more than an additional 10 minutes...people would not be wanting to do it...The providers are not going to want to order it. The nurses are not going to want to order it. It needs to be a quick thing that doesn't really significantly impact anything.” (P3)*
Cost	*“how do you compensate a physical therapist to be there 24/7, 365? We're probably busiest at 7:00 until midnight and then weekends, holidays, that kind of thing (P1).”* *“A huge part of it is money. So, the more patients you see, the more money the department gets, right? If you are holding patients to get the nonpharmacologic therapies done, that is less patients that you can ultimately see. And the more patients that then pile up in the waiting room” (P7)*

Pain management approaches vary based on patient presentation and provider preferences. For acute traumatic injuries, providers control pain directly with opioids. In contrast, for patients with chronic pain, a non-opioid approach is preferred. Policies also shape how opioids are prescribed. However, the absence of consistent ED-specific pain management guidelines compels some providers to avoid opioids altogether. Multimodal approaches are used to curtail a patient’s opioid regimen. For example, providers acknowledged potential benefits from nonpharmacological strategies. Physical therapy, cognitive-behavioral therapy, and counseling are thought to be helpful for some patients and could even prevent unnecessary admissions. However, while benefits of certain mind-body strategies were acknowledged, concerns were raised about whether these interventions lack compatibility with ED environments: “.*..I'm just picturing someone trying to do that in our insanely hectic ED and I just can't imagine. I'm sorry, I didn't mean to laugh. It's just hard to imagine blocking out all the noise and chaos in there to try to do [meditation and things] (P2).”*

Limited institutional resources are another significant challenge to implementation. Institutional constraints are associated with physical space, time, and cost. Strategies such as acupuncture are not as high priority for a room or bed as other competing needs in the ED. Limited time during encounters and the 24-hour nature of the ED are seen as barriers related to cost. For example, extending time for patients receiving physical therapy or counseling impacts the number of other patients that can be seen.

### Professional beliefs about nonpharmacological interventions

Table 3 contains illustrative quotes related to professional beliefs. Providers expressed having little formal training in medical school on nonpharmacological interventions for pain, leaving them unfamiliar with how to use or recommend them in practice. Other providers relied on personal or professional experiences to guide their belief, or they looked to the scientific evidence. Generally, providers felt some nonpharmacological strategies could be valuable additions to pain management in the ED. Specifically, since most patients were considered not physically active, a physical therapist could be a helpful resource for home exercise. Additionally, brief counseling or prayer could address a pressing need around comorbid mental health issues or spiritual needs. These approaches could expand the range of options for patients: *“…when the patient’s already on multiple pain medicines and presenting with worsening pain, your tool set becomes smaller and smaller… so I think the usefulness comes into play where there’s just an additional tool in my toolbox (P6).”*

**Table 3:** Professional beliefs about nonpharmacological pain interventions.

Provider knowledge	
Professional training	*“… I trained 10 years ago and during my residency there was this huge push to just prescribe, prescribe, prescribe. You were a bad doctor if you did not acutely treat people's pain…(P1).”* *“In medical school, no one sits us down and says, “This is what an acupuncturist is. This is what they do. This is what they'd be good for, for your patients. Or a chiropractor does these particular techniques, this would be good.” So, we don't really have exposure to those things...” (P6)*
Personal and professional experience	*“I have personally found that not all physical therapists are as helpful as others...some unfortunately just kind of check the boxes. Seem to pass you off. So, you need a motivated physical therapist.” (P1)* *“I've had to take physical therapy for rehab after surgeries, and I know the benefits of it...over time, it will ultimately help with their pain, but they need to continue with it (P3).”* *“I think that the explanations behind what they (chiropractors) do is not scientific. I don't think that the explanation is very scientific necessarily, but I do find that at least some studies that I've seen, that patients have had benefit from chiropractic care.” (P4)*
Scientific evidence	*“...there's no good nonpharmacologic interventions out there that have been shown to improve outcomes...I think that's the problem. There's not a lot of available data.” (P8)* *“I am not familiar all that much with acupuncture overall, although I know that there is certainly evidence that it seems to work.” (P9)*
**Perceived value**	
Value as an adjunct tool	*“I think some counseling that was fairly targeted and reasonably brief could be pretty helpful...We have such a huge mental health problem, and we have a lot of homeless patients, a lot of patients that have chronic psychiatric conditions.” (P4)* *“I think physical therapy is absolutely fantastic to have in the emergency department or to have available as adjunct to the emergency department. I think there's a lot of circumstances in which that's proven to improve outcomes…” (P9)*
Specific preference	*“Honestly, it probably would help a lot of people. And someone, if they were asked by every nurse, “Would you want someone to come pray and talk with you?” Or like, “We're getting ready to discharge you. Would you want someone to come pray with you and provide you any spiritual support?” I bet you multiple people would say yes. I mean honestly, I feel like that would actually probably be beneficial.” (P3)*
**Safety or efficacy concerns**	
Adverse outcomes	*“…I have a lot of negative feedback from patients regarding physical therapy, so it's not always my go to… it really depends on what a pain issue is, whether physical therapy is discussed or not. Oftentimes I will throw it in there as a possibility…” (P4)*
Specific to chiropractic care	*“Essentially, we've had multiple patients who've had vertebral dissections from neck manipulation, so I really caution patients when it comes to that.” (P4)* *“….when I see people, especially elderly people, going to these chiropractors and having these manipulations performed, I certainly worry from an allopathic side that it could be inducing some more harm than would be the potential to cause good, particularly with neck manipulations...(P6)* *“... I think my general feelings are that sometimes chiropractors are trying to step a little bit beyond that role. Saying that they could cure all kinds of different things with manipulation.” (P7)*

These perceived benefits were offset by safety or efficacy concerns. Most often, chiropractic care raised red flags to providers, including overextending their role and providing non-evidence-based care for conditions unlikely to benefit from manipulation.

### Patient characteristics as a modifying factor

Table 4 contains illustrative quotes related to patient and intervention characteristics as modifying factors. Providers expressed the patient’s expectations, financial situation, insurance status, time demands, and lack of primary care physicians are barriers to nonpharmacological interventions. Some providers shared concerns about bringing up aspects related to psychology. For example, providers felt patients want their pain situation to be acknowledged and if they are not careful, patients may feel dismissed. Providers also noted psychosocial factors including the patient’s worldview and beliefs can influence their interest in mind-body strategies. Some providers felt if they endorsed or shared their experience with an intervention or had the patient try it during the ED visit, it could help with patient engagement. However, the ED context makes this challenging: *“You try to establish a trusting, kind of doctor/patient relationship, but it's hard to really develop firm trust between the patient and the physician if they only meet you once or twice. Yeah, I don't know if that's enough time to be able to say, “I think you should get an acupuncture.” That's probably a little bit of a hill to climb, for a lot of patients (P4).”*

**Table 4:** Modifying factors of patient and intervention characteristics.

Patient Characteristics	
Resources	*“And a lot of patients can't take off days, if they miss the day of work, they're not getting paid. And so, then they're not going to go to a physical therapy appointment that they have to pay for and then miss another day of work that they are not getting paid for.” (P1)* *“So many patients… don't end up having insurance… don't have a primary care doctor. So, I don't even know how easy it would be for them to get physical therapy, but I still say that they should (P3).”* *“The question is how motivated are they to go beyond something like just taking a pain medication for what’s going on? It depends, I think, on their lives, and if they have time for that...” (P4)*
Psychosocial factors	*“It can be hard… patients have certain expectations about their pain. To even suggest a semantic or psychological component can be problematic… Some are open and ask, ‘What else can I do?’… but more often you have to be careful, or they may perceive you’re suggesting their pain is not real (P1).”* *“I think there's a misunderstanding what mindfulness is… If it's brought up together with yoga or meditation, then I do think that there's a certain number of the populations that's going to ascribe that to Eastern religion… others would just think it's voodoo, but then there's also the group that I think they don't have the time, or the motivation, or the finances…(P4).”* *“...clinicians have to believe in it, in order to discuss it with patients, so I think you'd have much broader acceptance if there is more belief on the part of clinicians that [it] worked.” (P5)* *“I think there's also a lot of these folks, especially with chronic pain, are also dealing with psychological and maybe some psychosocial issues as well...They just want to be acknowledged that their pain is real and that's unbearable, but then teaching them how to cope with pain is another strategy. I wish we did a better job in terms of pain management.” (P8)* *“… unless you do the intervention in person while they're there, I think you're not going to get a lot of people following through... these chronic pain patients, my impression is that they don't follow up with their appointments, especially if it's not going to be what they want… I think people are very, in general, skeptical of nonpharmacologic means of controlling pain.” (P8)*
Pain severity	*“There's some patients that are so desperate, they'll try anything, right?... But then you got those who I think … they're there just to get narcotics. And I don't think they'll be willing to hear anything about mindfulness or acupuncture unless they get the pill that they want... they'll say, Nope, that's not for me, and they'll leave.” (P8)*
**Intervention characteristics**	
Adaptability	*“…maybe when the acupuncturist just pulls them out of the CFU, does their thing and then puts them back in the CFU, I think that might be an idea. So that way they're not technically taking a bed in the emergency department, but they're still being attended to and being intervened upon.” (P8)*
Complexity	*“Is it something that would be an easy resource like, can I click a button and make this happen? Do I have to put in multiple phone calls? Do I have to put in multiple pages? Do I have to wait for someone to call me back? Those are just so many additional steps (P3).”* *“... if I could see that patient quickly and say, ‘...Here's an acupuncture clinic you can go to in the hospital.’ And I can discharge them there, then I think that would be awesome...but if they just went and disappeared for an hour or two, and then I had to follow up with them and they come back, that's really difficult with our flow to keep track of (P7).”* *“...if they (patients) then had a lot more beyond that referral, then the time stretch becomes probably more of a challenge for a provider's time and an effort.” (P9)*
Evidence	*“I haven't done any mind, body stuff, partly because I don't have a good sense of the data on that, but if there was a way to be able to connect a patient with it and I felt that there was some reasonable data showing some efficacy, then, I could include that in my skill set... or toolbox when approaching patients.” (P5)* *“… I think a lot of folks are motivated by evidence to consider offering something or to bring that to folks. In a situation where it's not evidence-based or we don't have the evidence yet, and that's the goal, then the other thing is, well, is it going to hurt them?...” (P9)*

The severity of pain was perceived as strongly shaping a patient’s willingness to try nonpharmacological approaches. Some providers felt patients in acute distress would be less open to alternatives and more focused on immediate pain relief through medication. Others felt patients would be willing to try something out of desperation.

### Intervention characteristics as a modifying factor

Providers emphasized interventions need to be simple and fit the constraints of the ED setting. Extending time or disrupting flow in the ED was a notable concern. Providers worried about complexity, noting the added work of arranging insurance approvals or coordinating outside services. Patients leaving the department for nonpharmacological services but still under the ED provider’s care is another concern. Providers expressed how strategies can be adapted to the ED, particularly around location, disposition time, and securing appointments. Helpful options include offering services early during their stay or while the patient is waiting to be seen or in an admitted area (i.e., POD): *“It depends when the provider is ordering the service, if we know this patient's going to [have] an issue with pain control...if they order at the beginning of their stay, they still have other things that we're waiting on, so it's not going to be a time limiting factor...(P6).”* Additionally, if patients can be treated in a co-located space and brought back to the ED, then this may reduce concern of having to track them down.

Finally, providers emphasized the need for clear evidence before recommending new approaches. Several providers said they would be more likely to recommend mind-body strategies if they were aware of published data demonstrating benefits for their patients, such as improvements in satisfaction, pain scores, return ED visits, or lower medication use. Other providers wanted to see evidence of safety, adherence, or long-term outcomes.

### Process of implementation and engagement

Table 5 contains illustrative quotes related to the process of implementation and engagement. Implementing nonpharmacological interventions in the ED would require thoughtful planning, education, and support. Providers need clear communication and guidance for successful implementation. One provider expressed *“…Having bullet points there so that we know what the resources are, and how to put in the order, how to do it, so that… we have it for the first time that we do it. And then we can tell other people, “That's where it is. That's how you do it.” And then in the future, obviously if you forget how to do it, it's there. I think that that is the most realistic thing (P3).”* Education and training would need to target physicians, advanced practice providers, and nurses as they are all part of the workflow. Education on workflow integration and scientific underpinnings were expressed as important for enhancing engagement. Additionally, providers felt identifying an ED provider champion was necessary. Finally, providers expressed leadership support was important for addressing any space requirements, funding needs, or expanding personnel in the ED.

**Table 5:** Process of implementation and engagement.

**Process of implementation**	
Planning for workflow integration	*“… I think if we know that we can send somebody the same day physical therapy, we're probably going to use it, We have some nurse practitioners who are working on fast track area... And they probably see the highest majority of low back pain patients or benign muscular cell complaints. So they would certainly need to be involved in the process… nursing would be important in terms of workflow of just communication, if, okay, we put you in for an appointment now… and make sure that the check-in process was easy and the communication process was appropriate.” (P1)* *“…I think a lot of times, it's an afterthought… I think working with operations to understand how to do that (psychological, psychiatric, mind/body interventions) would be important (P5).”*
Provider education	*“…there would need to be some kind of discussion about the scientific underpinnings of it, and where it would be successful and what kind of complaints it would be helpful [for]. You'd have to kind of sell it to faculty and staff, I think (P4).”* *“Provided it (psychological intervention) fits in with the workflow, then yes, I think that would be important. But I think that clinicians need to know when and how to potentially engage them (social work) because I don't think that's always understood. But yes, I think there's a role for it because I think when it comes to pain, that it's not always that there's actually pain. I think it's other potential factors influencing it as well.” (P5)*
**Engagement**	
Provider-level engagement	*“I think that having a champion in the ED whether it be social work or a provider or a nurse who's willing to champion this and move it forward I think is certainly a feasible thing. It's just a matter of finding that person.” (P6)* *“I think a champion in the ED is necessary for any change. So, this is coming down the pipe there's going to need to be somebody who is the champion. And I think honestly, some of these things are one person is showing that it's working and that they're able to do it effectively with the flow and then kind of organically other people can gather onto that.” (P7)*
Support from leadership	*"If you need physical space, then you need to have somebody at the higher levels of the hospital who can either give you that space from some other area or fund the development of that space.” (P1)* *“I think it depends if you're planning on having a physical therapist actually in the emergency department or not, if you were then you definitely need the medical director of the specific emergency department, because they would work with you on implementation, workflow.” (P1)*

## Discussion

This qualitative study explored providers’ perspectives on nonpharmacological pain management approaches in the ED. We had four key findings. First, providers expressed enthusiasm for multimodal pain care, especially for patients with chronic pain or a history of opioid use. Second, while they expressed enthusiasm, they also acknowledged practical and organizational barriers may limit routine use. Third, nonpharmacologic strategies such as physical therapy and brief counseling were viewed as helpful, but providers perceived that stronger evidence of efficacy for important ED outcomes is needed prior to implementation. Finally, leadership support and feasible pathways and interventions matching the pace and context of the ED are considered essential for future integration. Together, these findings highlight the promise of nonpharmacological pain management and the complexity of implementation in the emergency care environment.

Our findings are consistent with prior literature suggesting ED providers support evidence-based multimodal pain management but face multiple barriers to putting it into practice [40]. Some barriers reflect broader implementation challenges affecting a variety of healthcare settings, while others stem from the ED-specific context or intersect with the unique workflow and patient care demands. Environmental barriers to nonpharmacological pain management noted by our study and others include limited time in the ED, inadequate space, high patient volume and overcrowding, staff shortages, and lack of access to relevant resources [40,41]. Management of patients with chronic pain is challenging, often requiring more provider-patient time, follow-up, and attention to psychosocial and behavioral factors [42,43]. The ED is built for rapid assessment, decision making, and disposition, and not the longer duration holistic care patients with chronic pain often require. Gauntlett-Gilbert et al. [44] also highlighted the mismatch of patient and provider factors influencing chronic pain care in the ED. For example, patients may misunderstand what the ED can offer and arrive expecting immediate and dramatic pain relief. Consistent with our findings, providers feel unprepared and have limited options to fully manage chronic pain in the ED, and have competing priorities such as shifting their attention to more urgent situations or the pressure to see more patients.

Building on past studies focused on provider perspectives of medication use for chronic pain management [42], we directly explored how ED providers view nonpharmacological strategies for pain. Providers viewed approaches such as cognitive behavioral therapy or counseling as potentially useful for addressing psychological and social factors, especially for patients with chronic or recurring symptoms. At the same time, they expressed uncertainty about the strength of evidence of these treatments specifically for patients seeking ED care or the practicality of using them in the ED environment. Lack of familiarity with mind-body strategies stems from limited education in their medical training. Practitioners in other non-ED settings have reported similar needs and advocated for a move towards “psychologically-informed” care [45–47]. In our study, some providers noted their reliance on personal or professional experiences to make decisions regarding nonpharmacologic strategies with their patients. There is opportunity to educate ED providers on the evidence of nonpharmacological interventions for patients in non-ED settings, while also advancing work on the feasibility and efficacy of specific strategies such as brief educational and/or counseling interventions that could be effective for patients presenting to the ED.

Our findings point to several opportunities for improving how nonpharmacological pain treatments are introduced and sustained in emergency settings. Successful implementation will depend on understanding broad healthcare and ED-specific barriers to nonpharmacological pain care and identifying possible strategies to mitigate these barriers. The barriers identified in this study cut across multiple CFIR constructs and require strategies at multiple levels. Furthermore, our conceptual model, informed by CFIR and other frameworks, was developed to assist in identifying key factors that should be considered when targeting nonpharmacological intervention implementation in the ED. For example, successful implementation would require leadership and administrative support (institutional context and process barriers), feasible workflows including EHR prompts or brief scripts (intervention characteristics and process barriers), and provider education to build familiarity with nonpharmacological interventions for pain (institutional context, provider characteristics, and process barriers). Additionally, streamlined referral systems (institutional context and patient characteristics barriers) to outpatient programs or clinical services near the ED may help support continuity between acute and ongoing pain care. Another approach may involve embedding personnel directly into the ED setting. Co-location of intervention services was suggested as a possible strategy in the current study, however proper selection of care approaches needs to be considered for receptivity, adaptability, and fit for the ED. For example, brief spiritual consultation or counseling was well regarded, while chiropractic care was scrutinized by our participants. Recent work by Kim and colleagues [48] have shown efficacy of physical therapy in the ED for patients with low back pain. In their study of an ED-embedded pain coach educator program, LeLaurin et al. [49,50] used four primary strategies for implementation: 1) electronic health record modifications, 2) ongoing training and promotion activities, 3) clinical champions across disciplines, and 4) clinician recognition for top program utilizers. Providers in our study recommended similar tactics for integrating nonpharmacological approaches for pain into the ED.

While our study had many strengths related to its detailed qualitative interviews, there were limitations. This study was conducted at a single academic medical center located in the southeastern United States. As some findings are likely transferable, including the recognition of broad barriers to pain care such as limited time, space, or chronic pain complexity, other barriers may be unique to our ED setting. These context-specific factors may include characteristics of our patients, payer mix, institutional culture, and access to various conventional or complementary nonpharmacological health programs. The sample included nine ED physicians from the same institution. A larger sample may offer additional insight. Additionally, perspectives from non-physician staff, such as nurses and advanced practice providers, were not captured. Non-physician staff may have different views that would offer additional insight regarding nonpharmacological pain management in the ED. For example, non-physician staff may be more familiar with and likely to prescribe non-opioid regimens for back pain, especially in states restricting their prescribing ability. Future work should include multiple ED sites and a broader mix of provider roles to test whether these themes are consistent with the current study. Social desirability may also have shaped some responses and participants may have framed their views to match current expectations for good practice. Despite these limitations, the in-depth interviews offer valuable insight into ED physician perspectives of nonpharmacological pain management approaches.

## Conclusion

Overall, this study suggests ED physicians support a multimodal approach to pain care but face broad and ED-specific implementation barriers related to time, workflow, knowledge, and institutional resources. Addressing these barriers will require leadership support, provider training, and building efficient workflows fitting the pace of the care setting. These findings point to a clear need for nonpharmacological treatment models for pain working within the scope and constraints of emergency care. Future research will consider diverse interprofessional perspectives to further refine and operationalize the proposed conceptual framework.

## Supporting information

S1 File Interview guide questions. (DOCX)

S2 File Hierarchical coding system. (XLSX)

## References

[1] Chang H-Y, Daubresse M, Kruszewski SP, Alexander GC. Prevalence and treatment of pain in EDs in the United States, 2000 to 2010. Am J Emerg Med. 2014;32(5):421–31. doi: 10.1016/j.ajem.2014.01.015 24560834

[2] Cordell WH, Keene KK, Giles BK, Jones JB, Jones JH, Brizendine EJ. The high prevalence of pain in emergency medical care. Am J Emerg Med. 2002;20(3):165–9. doi: 10.1053/ajem.2002.32643 11992334

[3] Mura P, Serra E, Marinangeli F, Patti S, Musu M, Piras I, et al. Prospective study on prevalence, intensity, type, and therapy of acute pain in a second-level urban emergency department. J Pain Res. 2017;10:2781–8. doi: 10.2147/JPR.S137992 29263692 PMC5732548

[4] Giffin NA, Liedtke R, Poonai N, Holmes A, Wright B, Ali S. Prevalence of pain-related presentations in Canadian pediatric emergency departments. CJEM. 2024;26(9):650–7. doi: 10.1007/s43678-024-00729-w 38922497

[5] Small RN, Shergill Y, Tremblay S, Nelli J, Rice D, Smyth C, et al. Understanding the Impact of Chronic Pain in the Emergency Department: Prevalence and Characteristics of Patients Visiting the Emergency Department for Chronic Pain at an Urban Academic Health Sciences Centre. Can J Pain. 2019;3(1):106–13. doi: 10.1080/24740527.2019.1587290 35005399 PMC8730626

[6] Fellner A, Kim HS. Usual care for low back pain at United States emergency departments, 2016-2022. Ann Emerg Med. 2025. doi: 10.1016/j.annemergmed.2025.06.005 40650645 PMC12258925

[7] Bernard AM, Wright SW. Chronic pain in the ED. Am J Emerg Med. 2004;22(6):444–7. doi: 10.1016/j.ajem.2004.07.026 15520937

[8] Lam CN, Lui R, Allen R, Abramson T, Johnson E, Burner E. Hurting for options: emergency department utilization for chronic pain in a safety-net hospital. Acad Emerg Med. 2025. doi: 10.1111/acem.70127 40847459

[9] QuickStats: Percentage of emergency department visits for pain at which opioids were given or prescribed, by patient age and year - National hospital ambulatory medical care survey, United States, 2010-2020. MMWR Morbidity and mortality weekly report. 2022;71(51–52):1634. doi: 10.15585/mmwr.mm715152a4 36580438 PMC9812440

[10] Gottlieb M, Bernard K. Epidemiology of back pain visits and medication usage among United States emergency departments from 2016 to 2023. Am J Emerg Med. 2024;82:125–9. doi: 10.1016/j.ajem.2024.06.020 38905718

[11] Kamper SJ, Logan G, Copsey B, Thompson J, Machado GC, Abdel-Shaheed C, et al. What is usual care for low back pain? A systematic review of health care provided to patients with low back pain in family practice and emergency departments. Pain. 2020;161(4):694–702. doi: 10.1097/j.pain.0000000000001751 31738226

[12] Walkerly A, Neugebauer RE, Misko B, Shively D, Singh S, Chahda B, et al. Prevalence, predictors and trends of opioid prescribing for lower back pain in United States emergency departments. J Clin Pharm Ther. 2021;46(3):698–704. doi: 10.1111/jcpt.13324 33314253

[13] Chou R, Hartung D, Turner J, Blazina I, Chan B, Levander X. Opioid Treatments for Chronic Pain. Rockville (MD): Agency for Healthcare Research and Quality; 2020. 32338848

[14] Coombs DM, Maher CG, Collett M, Mathieson S, Abdel Shaheed C, Lin C-WC, et al. Continued opioid use following an emergency department presentation for low back pain. Emerg Med Australas. 2022;34(5):694–7. doi: 10.1111/1742-6723.13979 35441464

[15] Chen Q, Maher CG, Han CS, Abdel Shaheed C, Lin C-WC, Rogan EM, et al. Continued Opioid Use and Adverse Events Following Provision of Opioids for Musculoskeletal Pain in the Emergency Department: A Systematic Review and Meta-Analysis. Drugs. 2023;83(16):1523–35. doi: 10.1007/s40265-023-01941-1 37768540 PMC10624756

[16] Freiermuth CE, Foster JA, Manandhar P, Arulraja E, Erkanli A, Pollack CV, et al. Opioid Treatment Is Associated with Recurrent Healthcare Visits, Increased Side Effects, and Pain. West J Emerg Med. 2024;25(6):875–82. doi: 10.5811/westjem.18380 39625757 PMC11610737

[17] Ginsberg Z, Ghaith S, Pollock JR, Hwang AS, Buckner-Petty SA, Campbell RL, et al. Relationship Between Pain Management Modality and Return Rates for Lower Back Pain in the Emergency Department. J Emerg Med. 2021;61(1):49–54. doi: 10.1016/j.jemermed.2021.01.022 33637379

[18] Hayden JA, Ellis J, Asbridge M, Ogilvie R, Merdad R, Grant DAG, et al. Prolonged opioid use among opioid-naive individuals after prescription for nonspecific low back pain in the emergency department. Pain. 2021;162(3):740–8. doi: 10.1097/j.pain.0000000000002075 32947539

[19] Heard K, Ledbetter CM, Hoppe JA. Association of Emergency Department Opioid Administration With Ongoing Opioid Use: A Retrospective Cohort Study of Patients With Back Pain. Acad Emerg Med. 2020;27(11):1158–65. doi: 10.1111/acem.14071 32609923 PMC8079173

[20] Qaseem A, Wilt TJ, McLean RM, Forciea MA, Clinical Guidelines Committee of the American College of P. Noninvasive Treatments for Acute, Subacute, and Chronic Low Back Pain: A Clinical Practice Guideline From the American College of Physicians. Annals of Internal Medicine. 2017;166(7):514–30. doi: 10.7326/M16-2367 28192789

[21] US Department of Health. Pain management best practices interagency task force report: updates, gaps, inconsistencies, and recommendations 2019 [November 4, 2025]. Available from: https://www.hhs.gov/sites/default/files/pain-mgmt-best-practices-draft-final-report-05062019.pdf

[22] Machado GC, Ghinea N, Rogan E, Day RO, Maher CG. Emergency department care for low back pain: Should we adopt recommendations from primary care guidelines? Emerg Med Australas. 2020;32(5):890–2. doi: 10.1111/1742-6723.13593 32743874

[23] Bolton D. Looking forward to a decade of the biopsychosocial model. BJPsych Bull. 2022;46(4):1–5. doi: 10.1192/bjb.2022.34 35781123 PMC9768524

[24] Kooijmans ECM, Hoogendijk EO, Drapała N, Antonenko O, Burchell GL, Barańska I, et al. Defining and Categorizing Nonpharmacologic Interventions in the Older Population: A Systematic Review. J Am Med Dir Assoc. 2025;26(1):105306. doi: 10.1016/j.jamda.2024.105306 39424279

[25] Wang Y, Aaron R, Attal N, Colloca L. An update on non-pharmacological interventions for pain relief. Cell Rep Med. 2025;6(2):101940. doi: 10.1016/j.xcrm.2025.101940 39970872 PMC11866493

[26] Eucker SA, Foley S, Peskoe S, Gordee A, Risoli T, Morales F, et al. Willingness to use nonpharmacologic treatments for musculoskeletal pain in the emergency department: a cross-sectional study. Pain Rep. 2022;7(5):e1027. doi: 10.1097/PR9.0000000000001027 35999902 PMC9387978

[27] Flynn SB, Gordee A, Kuchibhatla M, George SZ, Eucker SA. Moving toward patient-centered care in the emergency department: patient-reported expectations, definitions of success, and importance of improvement in pain-related outcomes. Acad Emerg Med. 2021. doi: 10.1111/acem.14328 34358379

[28] Nunn ML, Hayden JA, Magee K. Current management practices for patients presenting with low back pain to a large emergency department in Canada. BMC Musculoskeletal Disorders. 2017;18(1):92. doi: 10.1186/s12891-017-1452-1 28228138 PMC5322663

[29] Tong A, Sainsbury P, Craig J. Consolidated criteria for reporting qualitative research (COREQ): a 32-item checklist for interviews and focus groups. Int J Qual Health Care. 2007;19(6):349–57. doi: 10.1093/intqhc/mzm042 17872937

[30] Saunders B, Sim J, Kingstone T, Baker S, Waterfield J, Bartlam B, et al. Saturation in qualitative research: exploring its conceptualization and operationalization. Qual Quant. 2018;52(4):1893–907. doi: 10.1007/s11135-017-0574-8 29937585 PMC5993836

[31] Hemmler VL, Kenney AW, Langley SD, Callahan CM, Gubbins EJ, Holder S. Beyond a coefficient: an interactive process for achieving inter-rater consistency in qualitative coding. Qualitative Research. 2020;22(2):194–219. doi: 10.1177/1468794120976072

[32] Azungah T. Qualitative research: deductive and inductive approaches to data analysis. Qual Res J. 2018;18:383–400.

[33] Fereday J, Muir-Cochrane E. Demonstrating Rigor Using Thematic Analysis: A Hybrid Approach of Inductive and Deductive Coding and Theme Development. International Journal of Qualitative Methods. 2006;5(1):80–92. doi: 10.1177/160940690600500107

[34] Tjora A. Qualitative research as stepwise-deductive induction. 1st ed. London: Routledge; 2018.

[35] Damschroder LJ, Aron DC, Keith RE, Kirsh SR, Alexander JA, Lowery JC. Fostering implementation of health services research findings into practice: a consolidated framework for advancing implementation science. Implement Sci. 2009;4:50. doi: 10.1186/1748-5908-4-50 19664226 PMC2736161

[36] Lehman BJ, David DM, Gruber JA. Rethinking the biopsychosocial model of health: Understanding health as a dynamic system. Soc Personal Psychol. 2017;11(8). doi: ARTNe12328

[37] Tramonti F, Giorgi F, Fanali A. Systems thinking and the biopsychosocial approach: a multilevel framework for patient-centred care. Syst Res Behav Sci. 2021;38(2):215–30.

[38] Golden SD, Earp JAL. Social ecological approaches to individuals and their contexts: twenty years of health education & behavior health promotion interventions. Health Educ Behav. 2012;39(3):364–72. doi: 10.1177/1090198111418634 22267868

[39] Stokols D. Translating social ecological theory into guidelines for community health promotion. Am J Health Promot. 1996;10(4):282–98. doi: 10.4278/0890-1171-10.4.282 10159709

[40] Burgess L, Theobald KA, Kynoch K, Keogh S. Assessment of barriers, supports, and context to implement best practice pain management in the emergency department: The IMPAINED study. Pain Manag Nurs. 2024;25(4):346–53. doi: 10.1016/j.pmn.2024.03.010 38825427

[41] Almutairi A, Coyer F, Keogh S, Hughes J. Factors influencing pain management in patients presenting to the emergency department: A mixed-method systematic review. Int J Nurs Stud. 2025;172:105214. doi: 10.1016/j.ijnurstu.2025.105214 40992019

[42] Wilsey BL, Fishman SM, Crandall M, Casamalhuapa C, Bertakis KD. A qualitative study of the barriers to chronic pain management in the ED. Am J Emerg Med. 2008;26(3):255–63. doi: 10.1016/j.ajem.2007.05.005 18358933

[43] McLeod D, Nelson K. The role of the emergency department in the acute management of chronic or recurrent pain. Australas Emerg Nurs J. 2013;16(1):30–6. doi: 10.1016/j.aenj.2012.12.001 23622554

[44] Gauntlett-Gilbert J, Rodham K, Jordan A, Brook P. Emergency Department Staff Attitudes Toward People Presenting in Chronic Pain: A Qualitative Study. Pain Med. 2015;16(11):2065–74. doi: 10.1111/pme.12844 26177229

[45] Astin JA, Goddard TG, Forys K. Barriers to the integration of mind-body medicine: perceptions of physicians, residents, and medical students. Explore (NY). 2005;1(4):278–83. doi: 10.1016/j.explore.2005.04.014 16781549

[46] Sierpina V, Levine R, Astin J, Tan A. Use of mind-body therapies in psychiatry and family medicine faculty and residents: attitudes, barriers, and gender differences. Explore (NY). 2007;3(2):129–35. doi: 10.1016/j.explore.2006.12.001 17362848

[47] Driver C, Kean B, Oprescu F, Lovell GP. Knowledge, behaviors, attitudes and beliefs of physiotherapists towards the use of psychological interventions in physiotherapy practice: a systematic review. Disabil Rehabil. 2017;39(22):2237–49. doi: 10.1080/09638288.2016.1223176 27635464

[48] Kim HS, Ciolino JD, Lancki N, Strickland KJ, Pinto D, Stankiewicz C, et al. A Prospective Observational Study of Emergency Department-Initiated Physical Therapy for Acute Low Back Pain. Phys Ther. 2021;101(3):pzaa219. doi: 10.1093/ptj/pzaa219 33351942 PMC7970627

[49] LeLaurin JH, Montague M, Salloum RG, Shiekh SS, Hendry P. Implementation of a novel emergency department pain coach educator program: First year experience and evaluation. Res Sq. 2023:rs.3.rs-2488709. doi: 10.21203/rs.3.rs-2488709/v1 36747798 PMC9901022

[50] LeLaurin JH, Montague M, Curtis ME, Salloum RG, Sheikh S, Hendry PL. Implementation of a novel pain coach educator program in a safety-net emergency department. Implement Res Pract. 2025;6:26334895251330511. doi: 10.1177/26334895251330511 40191386 PMC11970099

