# Supplementary material for: “Probably a Little Bit of a Hill to Climb”: A qualitative study of emergency department providers’ perceptions of nonpharmacological pain treatment
Source: PLoS One. 2026 Jun 1;21(6):e0350266. doi: 10.1371/journal.pone.0350266 (PMC13225396; doi:10.1371/journal.pone.0350266)
Supplement: S1 File — (DOCX) [file pone.0350266.s001.docx]

**S1 Appendix. Interview guide questions.**

**Questions on current pain management**

- How frequently do you encounter patients seeking pain management (i.e., one time per shift, every other shift, etc)?
  - How often do you encounter patients with chronic pain?
- How would you describe your current pain management approach?
  - *What guideline(s) do you follow?*
  - *What treatment(s) do you commonly prescribe or recommend?*
- How often have you prescribed or recommended nonpharmacological pain treatments (such as physical therapy, counseling, mindfulness, acupuncture) to ED patients?
  - *What aspects of these treatments do you feel are helpful for patients?*
  - *How do you communicate the benefits of these treatments to these patients?*
  - *What have you found is the most helpful information to provide to patients that may enhance their engagement with these treatments?*

**Questions on nonpharmacological pain treatments**

- What are your thoughts on the need for offering nonpharmacological pain treatments within the ED setting for acute and chronic pain?
  - *Should nonpharmacological pain treatments be seen as alternatives or complements to narcotic and non-narcotic pain medication?*
  - *What non-pharmacological treatments do you feel are most effective?*
  - *Are there treatments you feel are less effective?*
  - *How might embedding nonpharmacological pain treatments within the ED be effective?*
  - *What outcomes might be improved?*
- What patients do you feel would benefit most from these types of treatments?
  - *What patient characteristics would be important when thinking of who might benefit?*
  - *What clinical information would you want to know that is not routinely asked or assessed?*
- What types of nonpharmacological treatments would be acceptable to patients and feasible to offer?
  - Examples: physical treatments (PT, chiropractic, acupuncture, yoga), psychological-based (counselling, mindfulness), spiritual (prayer)
  - *What are your thoughts on nonpharmacological treatments started after ED discharge compared to these treatments provided within the ED?*
  - *What are your thoughts on remote telephone or internet-based treatments started after ED discharge?*
- How might nonpharmacological pain treatments be embedded within the current ED workflow?
  - *What training might ED providers need for implementing nonpharmacological pain treatments?*
  - *What communication or education strategies would be useful for recommending these treatments? Real-time Best Practice Alerts? Training as part of Tuesday conference? Both?*
  - *Are there clinical specialties that would be helpful to embed within the ED?*
  - *How might nonpharmacological pain treatments be combined with other strategies?*
  - *Are there departments, centers, or institutes that would be ideal to partner with for delivering these treatments?*
  - *What other opportunities might there be for embedding nonpharmacological pain treatment?*
- What barriers do you anticipate in implementing nonpharmacological pain treatments?
  - *What policies, incentives, or processes would limit the ability to implement nonpharmacological pain treatments?*
  - *What impact would there be to ED workflow?*
- What factors would facilitate implementing nonpharmacological pain treatments?
  - *What policies, incentives, or processes would enhance the ability to implement nonpharmacological pain treatments?*
  - *What changes to the ED workflow would enable implementation of nonpharmacological pain treatment?*
  - *What outcome metrics do you feel would need to be met to support embedding nonpharmacological pain treatment?*
